# Energetic Valorisation of Saltworks Bitterns via Reverse Electrodialysis: A Laboratory Experimental Campaign

**DOI:** 10.3390/membranes13030293

**Published:** 2023-02-28

**Authors:** Syed Abdullah Shah, Roberta Cucchiara, Fabrizio Vicari, Andrea Cipollina, Alessandro Tamburini, Giorgio Micale

**Affiliations:** 1 Dipartimento di Ingegneria, Università degli studi di Palermo, Viale delle Scienze ed. 6, 90129 Palermo, Italy; 2 ResourSEAs SRL, Viale delle Scienze ed. 16, 90128 Palermo, Italy

**Keywords:** brine, salinity gradient power, RED, salty water

## Abstract

Concentrated bitterns discharged from saltworks have extremely high salinity, often up to 300 g/L, thus their direct disposal not only has a harmful effect on the environment, but also generates a depletion of a potential resource of renewable energy. Here, reverse electrodialysis (RED), an emerging electrochemical membrane process, is proposed to capture and convert the salinity gradient power (SGP) intrinsically conveyed by these bitterns also aiming at the reduction of concentrated salty water disposal. A laboratory-scale RED unit has been adopted to study the SGP potential of such brines, testing ion exchange membranes from different suppliers and under different operating conditions. Membranes supplied by Fujifilm, Fumatech, and Suez were tested, and the results were compared. The unit was fed with synthetic hypersaline solution mimicking the concentration of natural bitterns (5 mol/L of NaCl) on one side, and with variable concentration of NaCl dilute solutions (0.01–0.1 mol/L) on the other. The influence of several operating parameters has also been assessed, including solutions flowrate and temperature. Increasing feed solutions’ temperature and velocity has been found to lower the stack resistance, which enhances the output performance of the RED stack. The maximum obtained power density (corrected to account for the effect of electrodic compartments, which can be very relevant in five cell pairs laboratory stacks) reached around 10.5 W/m^2^_cellpair_, with FUJIFILM Type 10 membranes, temperature of 40 °C, and a fluid velocity of 3 cm s^−1^ (as empty channel, considering 270 μm thickness). Notably, the present study results confirm the large potential for SGP generation from hypersaline brines, thus providing useful guidance for the harvesting of SGP in seawater saltworks all around the world.

## 1. Introduction

Brines produced from saltworks, desalination plants, and many other industrial activities contain high concentrations of total dissolved solids (TDS) and, depending on the process, can be released at a higher temperature than the environmental temperature. Directly discharging them into the ocean will not only deteriorate local marine ecology but also the wider surrounding environment [1,2].

Saltworks brine temperature is generally 30–40 °C [3], a kind of heat that can hardly be exploited and is generally classified as low-grade. In addition to the temperature gradient, a salinity one also exists in the saltworks, between the brine and seawater or locally available low-concentration streams such as wastewater treatment plant discharge.

Capturing this middle-temperature salinity gradient power (SGP) for energy generation might be essential for energy conservation, emission reduction, and coastal environmental preservation. Additionally, extracting renewable energy from brine can assist in achieving the seventh United Nations (UN) sustainable development goal (SDG7), affordable and clean energy.

At the moment, approaches for harvesting SGP include pressure retarded osmosis (PRO) [4,5], steam pressure energy (SPE) [6], and reverse electrodialysis (RED) [7,8,9,10]. Among them, the RED technology has drawn the attention of many researchers due to the advantages of no moving components, excellent reliability, and simple eradication of membrane fouling concerns [11].

**Figure 1:**
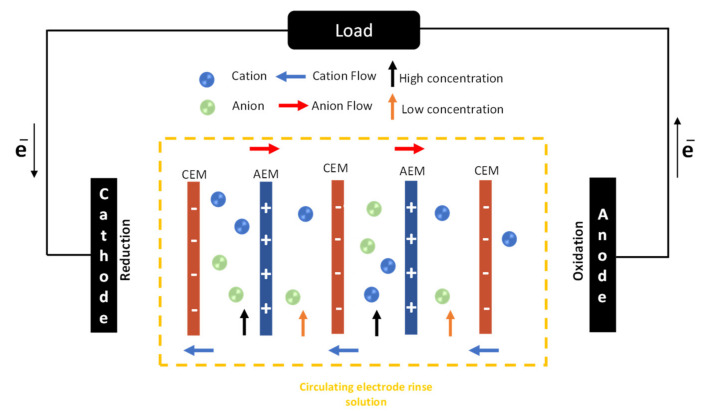
Schematic representation of the Reverse Electrodialysis RED (process). CEM: Cation exchange membrane; AEM: Anion exchange membrane.

As described in Figure 1, a RED device is composed of a series of anion exchange membranes (AEMs) and cation exchange membranes (CEMs) forming adjacent channels in which hypersaline and dilute solutions flow without contacting one another. The intermembrane distance is maintained by placing a polymeric spacer between AEM and CEM, which provides mechanical stability to the compartment. The AEMs/CEMs permit ion transport across them, enabling the concentration gradient existing between the solutions to be converted into an ordered flux of ions which can ultimately be used to sustain electrochemical processes at the electrode and produce an external net flow of electrons. In other words, RED can directly produce electrical energy by directing the ion exchange between two solutions.

RED has been used to convert seawater and freshwater into electricity [8,12,13], but also for wastewater treatment [14,15] and within a novel concept of a heat engine [16,17].

However, the RED industrial application is still limited by technical issues in producing high net power densities. To tackle this issue, researchers have tried many different approaches. As an example, Abdullah et al. demonstrated that a combination of multiple monovalent salts as feed solutions for RED could optimize power density, especially if compared with single salt solutions [7]. These findings are in line with those obtained by Micari et al., where the measured stack resistance for binary mixtures of salts was found to be lower than that of the pure salts in experiments, thus suggesting a potentially higher power density [18].

Net power density is primarily influenced by the energy spent for pumping the solutions across the stack, which depends on membrane fouling in time. In this regard, Cosenza et al. [14] tested a RED unit run over 25 days utilizing effluents from crude oil extraction processes. During the long-run tests, a maximum power density of 2.5 W m^−2^_cp_ was observed, and alternative anti-fouling techniques were investigated to manage the fouling occurrence.

Membrane fouling also inhibits ions migration as highlighted by Kang and co-workers, who found that maximum power density and open circuit potential could be increased by 19% and 9.4%, respectively, just by filtering out sediments from the feed [19].

The maximum power density ever reported with RED is the one from Daniilidis, who examined the effect of solution concentration and temperature on the performance of a RED stack, obtaining a maximum power density of 10.6 W m^−2^_cp_ and 13.4 W m^−2^_cp_ at 40 °C and 60 °C, respectively [20].

Concerning high salinity industrial streams, much work has been done for desalination brine management. As an example, Brauns and colleagues proposed a concept coupling RED with seawater desalination. After the desalination, the brine is released into a solar pond for additional concentration. Then, the concentrated brine and seawater are fed to the RED unit to generate electricity [21,22].

Less attention has been devoted to the valorization of bittern; the peculiar industrial exhaust solution is obtained after sodium chloride production in the saltworks.

The first 1 kW RED pilot plant fed with real solutions in a real environment was operated in a saltwork by Tedesco and colleagues, demonstrating the possibility of using bittern for as long as five months without losing performance [22].

**Figure 2:**
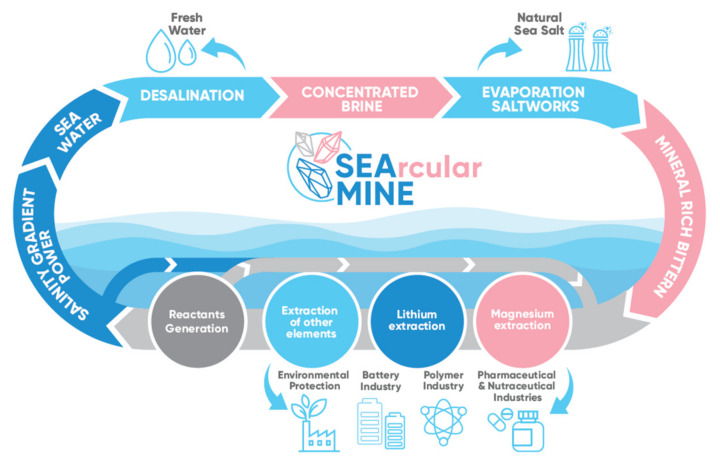
Schematic illustration of the SEArcularMINE project integrated process. Reprinted with permission from Ref. [23].

Noteworthy, when the very same stack was fed with synthetic solutions containing NaCl of the same TDS as the real one, the power density produced almost doubled its value because of the absence of divalent ions [22]. In the framework of the SEArcularMINE project, following the scheme of a novel approach designed and patented by ResourSEAs SrL, the presence of divalent ions affecting performance has been solved by placing the RED unit at the end of a mineral extraction sequence, reducing the presence of magnesium and calcium to negligible values (see Figure 2) [23].

In the present work, an extensive experimental campaign was conducted using a reverse electrodialysis unit. In particular, three different types of ion exchange membranes were tested for the recovery of salinity gradient energy using artificial brines mimicking the expected features of brines in a real application. More precisely, for the first time, RED units equipped with Fujifilm, Fumatech, and SUEZ IEMs were compared when operated with saltworks brine. The concentration and the temperature of the artificial feed were chosen based on the features of a real brine examined in the framework of the SEArcularMINE project. Furthermore, the effect of feed velocity and dilute solution concentration was also studied. This study could provide some guidance for harvesting salinity gradient energy from saltwork plants.

## 2. Experimental Setup and Procedure

### 2.1. Stack Configuration

Three RED stacks of the same size were built and assembled using different types of ion exchange membranes. In particular, the first RED stack was assembled with Fujifilm Type 10, AEMs/CEMs (Fujifilm Manufacturing Europe B.V., Tiburg, The Netherlands), a second stack was equipped with Fumatech AEM-FAB-PK-130/CEM-FKB-PK-130 (Fumatech BWT GmbH, Bietigheim-Bissingen, Germany). A third stack was built for further investigation using SUEZ membranes AEMs-AR103U/CEMs-CR67U (Suez, Paris, France). Membrane properties are reported in Table 1 for the sake of completeness. However, only some of them (i.e., perm-selectivity and electrical resistance) will be useful to discuss the collected results in Section 3. The three stacks were operated under the same experimental feed conditions. Each stack had an active area of 0.01 m^2^ per membrane. The RED unit (REDstack B.V., Sneek, The Netherlands) was equipped with five cell pairs separated by 270 μm spacers (Deukum, Frickenhausen, German), each cell pair is composed of one anion exchange membrane (AEM), one spacer, and one cation exchange membrane (CEM). Additional CEMs specifically selected for their high selectivity (Fumasep FKS-50, BWT GmbH, Bietigheim-Bissingen, Germany) were placed as shielding membranes next to each electrode compartment to prevent electrode rinse solution leakage into the high and low concentrations channels. 

The end plates of each unit are made of poly-methyl-methacrylate (PMMA) and hosted two Ru-Ir oxides coated titanium electrodes. The shielding membrane in the end compartments and endplate was separated using a silicon gasket, and the electrode compartments were filled with a woven spacer.

### 2.2. Experimental Setup

**Figure 3:**
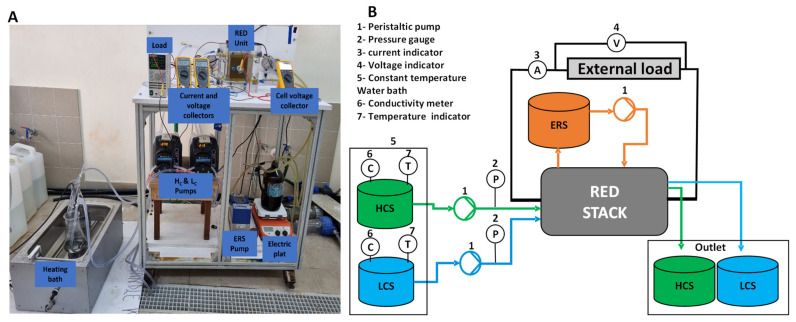
(**A**) Picture of the RED experimental setup. (**B**) shows the RED experimental diagram. HCS: high compartment solution; LCS: low compartment solution; ERS: electrode rinse solution.

The laboratory RED experimental setup is illustrated in Figure 3. Synthetic feed solutions were prepared using sodium chloride (NaCl 99.7% ChemSolute Renningen, Germany), dissolved in deionized water. Five low-salinity feed solutions and one synthetic brine were prepared according to the requirements of the experimental campaign (see Table 2). The feed solution was heated to a desired temperature with a heating bath, and a heated magnetic stirring plate (LLG, uniStirrer7, Am Hambuch, Germany) was used for the electrode rinse solution to keep the required temperature constant. The synthetic brine was prepared to closely match the real brine expected in the SEArcularMINE project. The ERS solution used for all the experiments contained 0.1 M of FeK_3_(CN)_6_/FeK_4_(CN)_6_ and 0.6 M NaCl as a supporting electrolyte.

A co-flow arrangement was adopted for the feed solutions. Peristaltic pumps were used to circulate feed and electrode rinse solutions to the stack (BT601S from Lead Fluid Technology, CO LTD, Hebei, China).

### 2.3. Experimental Procedure

Before each set of experiments, leakage tests were performed after the assembly of RED stack pumping only deionized water in high, low, and ERS compartments at a flow velocity of 1 cm s^−1^. For example, to quantify high channel leakage, water was circulated in low and ERS channels while the inlet of the high channel was maintained closed, and the outlet was left open. The percentage of leakage was determined by the volume (*V_mL_*)*_leak_* from the high compartment divided by the test duration (minutes) and flow rate (*Q* = 81 mL/min), as indicated in Equation (1) below. The test was carried out to ensure that the stack was assembled well and there was no internal mixing of the feed and ERS solution, Results of the leakage tests are shown in Table 3.

(1) $$Leakage\;\left(\%\right)=\frac{\left(V_{mL}\right)leak}{t\times Q}$$

Artificial sodium chloride solutions of various concentrations were used as the feed solutions. The concentrations of diluted solutions ranged from 0.01 M to 0.1 M with a constant concentrated solution of 5 M. Feed solutions were injected into the stack continuously at a fixed flow rate of 1, 2, and 3 cm s^−1^. The electrode rinse solution consisting of 0.1 M potassium ferricyanide K_3_Fe(CN)_6_, 0.1 M potassium ferrocyanide K_4_Fe(CN)_6_ (Honeywell Flute, Seelze, Germany), and 0.6 M sodium chloride (99.7% ChemSolute, Renningen, Germany), was recirculated through the electrode compartments of the stack. To avoid light exposure, the electrode rinse solution was contained in a black bottle. All the solutions were prepared in deionized water. The temperatures of the feed solutions (ranging from 20 to 40 °C) were controlled by a water bath and were continuously monitored using a temperature meter. Solution conductivity and pressure losses in the compartments were monitored by a conductivity meter (3320, Xylem, Weilheim in Oberbayern, Germany) and pressure gauges (Cewal, Camponogara, Italy). Multimeters (Fluke-175 True RMS, Everett, WA, USA) and an external load (BK Precision,8540, Yorba Linda, CA, USA) were employed to collect the polarization curves (i.e., current versus electric potential over the stack curves).

### 2.4. Performance Indicators

**Figure 4:**
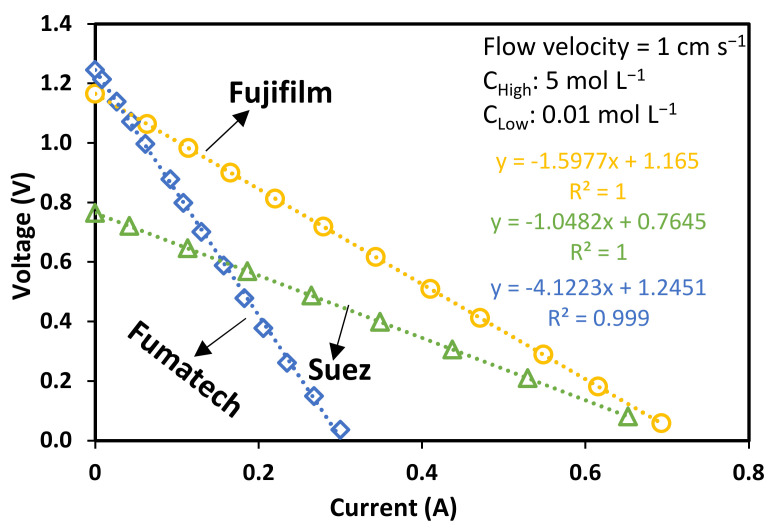
Polarization curves obtained with each IEMs set adopted in the study. The equation reported in the box shows, with reference to Equation (2), the values of *R_stack_* (angular coefficient), and *OCV* (y−intercept).

The performance of a RED unit can be expressed based on some performance indicators, which can be easily derived from the measured experimental information:

Stack electrical potential, $V_{stack}$:

(2) $$V_{stack}=OCV-I\;R_{stack}$$

where $V_{stack}$ is the output potential of the RED stack, *OCV* is open circuit voltage (measured when the external electrical load is disconnected), *I* is the electrical current (measured by an amperometer), and $R_{stack}$ is the electrical internal resistance of the stack which can be ideally expressed as:

(3) $$R_{stack}=R_{blank}+N_{cell}\;\left(AEM_{R}+CEM_{R}+High_{R}+Low_{R}\right)$$

where $R_{blank}$ represents the resistance of electrodic compartments and can be measured by assembling the RED stack with only one cation exchange membrane (Fumasep FKS-50, BWT GmbH, bietigheim, Germany) and feeding the electrode compartment with the above-mentioned rinse solution only. From Equation (2), it is clear how the internal resistance of the RED stack can be obtained from the slope of the output voltage (*V*) to current (*I*) curve (the so-called “polarization curve”), as shown in Figure 4 with three examples of polarization curves obtained with each of the membrane sets adopted in this study.

**Figure 5:**
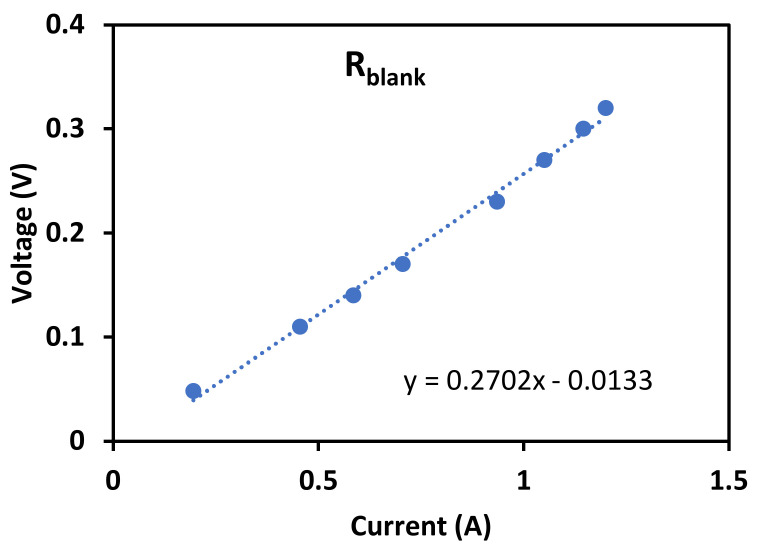
*R_blank_* as the slope between voltage and current under the experimental condition of 0.1 M FeK_3_(CN)_6_/FeK_4_(CN)_6_, and 0.6 M NaCl in the electrode compartment, 20 °C temperature, and 180 mL min−^−1^ of flow rate.

Figure 5, instead, shows the polarization curve with a one-membrane stack, to determine *R_blank_*, as the slope of the resultant linear trend, eventually determined to be 0.27 Ω.

The output power, *P* is given by:

(4) $$P=V_{stack}\times I\;$$

The power generation per unit cell pair area is defined as power density:

(5) $$PD=\frac{P}{NA}$$

where *N* is the number of cell pairs (5) and *A* is the active area of membranes (0.01 m^2^).

Once the value of $R_{blank}\;$is identified, the measured value of *PD* can be corrected ($PD_{corr}$) starting from Equation (5), in order to determine the power density corresponding to an ideally large number of cell pairs (e.g., in a full-scale stack), where the contribution given by electrode compartments to total stack resistance ($R_{blank}$) becomes negligible. In particular, the $PD_{corr}$ is calculated by subtracting the $R_{blank}$ from the stack resistance $R_{stack}$ and redetermining the main electrical variables according to [3]:

(6) $$PD_{corr}=\frac{OCV^{2}}{N\;A\;R_{load}{\left(1+\frac{R_{cells}}{R_{load}}\right)}^{2}\;}$$

where $R_{cells}=R_{stack}-R_{blank}$

Other than *PD_corr_* also the *PD_net,cover_*, was calculated, subtracting from the power density the power consumed by the pumps ($PD_{pump}$), theoretically estimated as the product of pressure drops and flow rate, then normalized by the total cell pair area:

(7) $$PD_{net,cover}=PD_{corr}-PD_{pump}$$

(8) $$PD_{pump}=\frac{\Delta P_{high}\times Q_{high}^{tot}+\Delta P_{low}\times Q_{low}^{tot}}{\;N.A}$$

Please, note that the power needed to heat the solutions to 30–40 °C is not taken into account because bitterns can achieve these temperature values in summer when they are discharged after the salt collection. Where $\Delta P_{high}$, $\Delta P_{low}$, and $Q_{high}^{tot}$, $Q_{low}^{tot}$ are, respectively, the pressure drop and flow rates both for the concentrate and dilute compartments.

The mean fluid velocity inside a single spacer filled compartment, *v,* has been calculated as:

(9) $$v=\frac{Q}{60\times N\times W\times T}$$

where Q is the flow rate of the feed solutions (mL min^−1^) in a single compartment, N is the number of cell pairs (5), W is the channel width (10 cm), and T is the spacer thickness (0.027 cm).

## 3. Results and Discussion

Membrane features, flow rates in the compartment channels, dilute solution concentration, and feed solutions temperature are the primary variables influencing the RED process [3,27]. Therefore, considering these parameters, experiments were implemented to study the influence of all these parameters on the performance of the RED stack, equipped with different ion exchange membranes.

### 3.1. Influence of the Temperature

**Figure 6:**
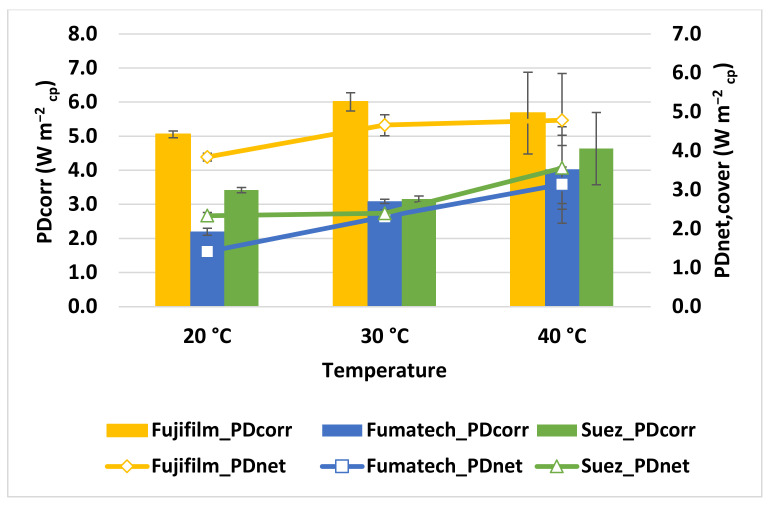
Influence of temperature on power density for the different IEMs under the experimental conditions of of *C_High_* = 5 mol L^−1^, *C_low_* = 0.06 mol L^−1^, and flow velocity of 1 cm s^−1^.

Figure 6 illustrates the variation of *PD_corr_* at different feed temperatures under the experimental conditions of *C_High_* = 5 mol L^−1^, *C_low_* = 0.06 mol L^−1^, and a flow velocity of 1 cm s^−1^.

As already reported in the literature [3,19,28], the temperature has a beneficial effect on power density, so that when increasing it from 20 °C to 40 °C, the *PD_corr_* increases for every membrane tested, though at different rates: from 5.0 W m^−2^_cp_ to 6.0 W m^−2^_cp_ for Fujifilm, from 2.2 W m^−2^_cp_ to 4.0 W m^−2^_cp_ for Fumatech, and from 3.4 W m^−2^_cp_ to 4.6 W m^−2^_cp_ for Suez stack.

**Figure 7:**
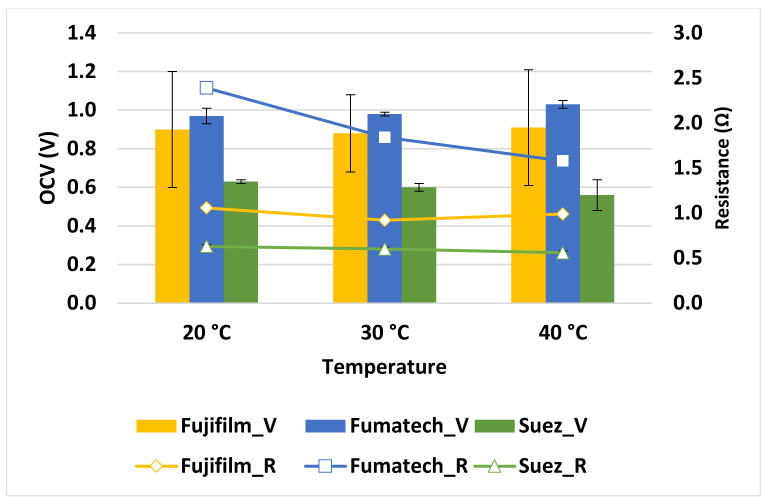
Variation of *OCV* and stack resistance with temperature under the experimental conditions of 5 mol L^−1^ *C_High_*, 0.06 mol L^−1^ *C_Low_*, and 1 cm s ^−1^ flow velocity.

In order to better explain the behaviour of the system, the trends of *OCV* and *R_stack_* have been monitored, as illustrated in Figure 7. Overall, the *OCV* of the stacks assembled with different IEMs has not been dramatically affected by the increase in temperature, whereas the internal resistance was differently reduced with the increase in temperature, likely due to the increase in ionic conductivity of the feed solutions and the membrane. In fact, the rise in temperature has been found to enhance the degree of swelling and enlarges the size of the pores in the membranes, which help ion migration through the membranes [29]. The effect of resistance reduction is particularly evident for the Fumatech stack, where there is a variation from 2.39 to 1.58 Ω, which resulted, eventually, in the increasing trend of PD for all membrane types [30].

#### 3.1.1. Influence of Dilute Solution Concentration

The influence of feed TDS has been evaluated at a fixed temperature of 20 °C by varying the concentration of the dilute solution for the stacks equipped with the three different membrane sets.

The concentration of the dilute solution (*C_Low_*) has been studied in the range of 0.01–0.1 mol L^−1^, which is what can be expected to be found at the outlet of a wastewater treatment plant [30].

**Figure 8:**
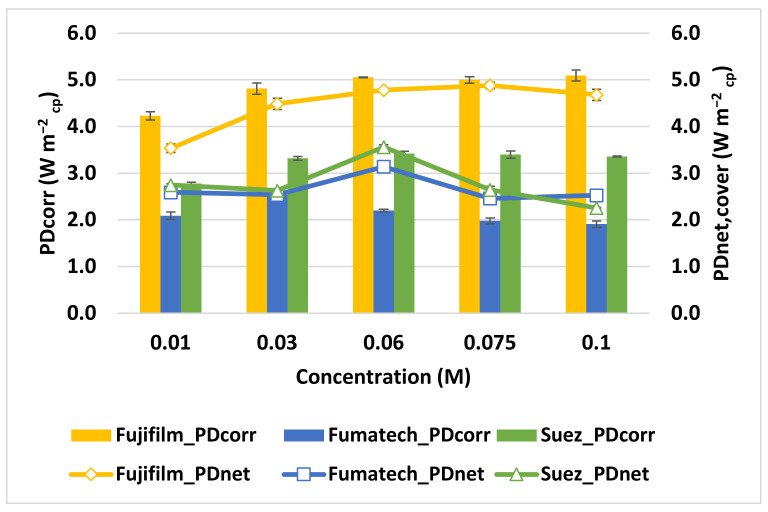
Influence of TDS concentration in the low salinity stream (*C_Low_*) on power density for the three different configurations. *C_High_* = 5 mol L^−1^, 20 °C temperature and flows velocity 1 cm s ^−1^.

It is clear how, for every concentration explored, the maximum *PD_corr_* for the RED is always obtained with the membranes provided by Fujifilm, followed by Suez membranes and Fumatech membranes (see Figure 8).

**Figure 9:**
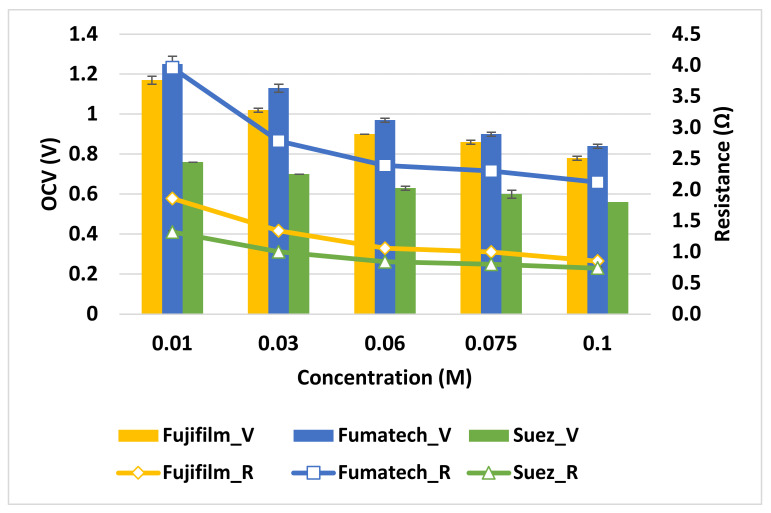
Variation of *OCV* and stack resistance with dilute concentration under the experimental conditions of 5 mol L^−1^ *C_High_*, 20 °C temperature and 1 cm s ^−1^ flow velocity.

On the other hand, the maximum *OCV* is obtained with Fumatech membranes (0.97 V) rather than Fujifilm (0.9 V) or Suez (0.63 V) (see Figure 9) These two discordant results can be explained by the different characteristics of the membrane under investigation; first of all, the average permselectivity of membranes *α_m_*, whose value determines the *OCV* according to Equation (10) [31].

(10) $$OCV=2N\alpha_{m}\frac{RT}{zF}\ln\left(\frac{a_{c}}{a_{d}}\right)$$

where *R* is the ideal gas constant (8.314 J(mol K)^−1^), *T* is the temperature (*K*), *z* is the valance number of ions (−), *F* is Faraday constant (96,485 C mol^−1^), and $a_{c}$/$a_{d}$ is the ionic activity of concentrated and diluted solution, respectively (mol m^−3^).

Hence, the *OCV* is significantly enhanced by the high permselectivity of both anion and cation Fumatech membranes and affected by the low values of SUEZ membranes (see Table 1).

The advantage of a high permselectivity comes with the cost of a higher membrane resistance, which results in an increase in stack resistance *R_stack_* (see Equation (3)) and, in turn, in a reduction of the total producible power *P* according to Equation (6).

Since Suez membranes have the lowest internal resistance among the three providers compared, even in the absence of a high *OCV*, they succeed in achieving a higher power density than the Fumatech stack.

As a consequence of this delicate equilibrium between membrane resistance and permselectivity, Fujifilm membranes, which have intermediate values of both parameters, result in the higher power density produced.

In the investigated range, increasing the concentration of the dilute compartment enhanced power production for Fujifilm and Suez stack, whereas in the last case (Fumatech membranes), the maximum power density was achieved for the intermediate concentration of *C_Low_* = 0.03 mol L^−1^ (see Figure 8).

This result can be interpreted as the different relative weights on stack resistance of the different membranes (see Equation (3)). Adopting SUEZ and Fujifilm membranes, dilute compartment resistance is always the limiting factor in power production for the explored *C_Low_* concentration range. Conversely, Fumatech membranes resistance is so high that it limits power production for every concentration greater than 0.03 M.

Fumatech membranes outperformed the other in terms of *OCV* at various concentrations, as shown in Figure 9**.** As expected, the general trend is a reduction of *OCV* moving *C_Low_* from 0.01 mol L^−1^ to 0.1 mol L^−1^ because of the reduction in the salinity gradient.

A particular behavior that can be observed in Figure 9 is the distance between the two curves representing the internal resistance of Fujifilm and SUEZ stacks, which is not constant and tends to become negligible at higher concentrations. This occurrence is probably due to the membranes behaving differently in the presence of highly concentrated solutions among both sides of CEM/AEM, which can result in swelling and ion sorption phenomena that affect the IEC of the IEMs [32] and ultimately contribute to lower the actual resistance of the thinner membranes.

#### 3.1.2. Influence of Feed velocity

**Figure 10:**
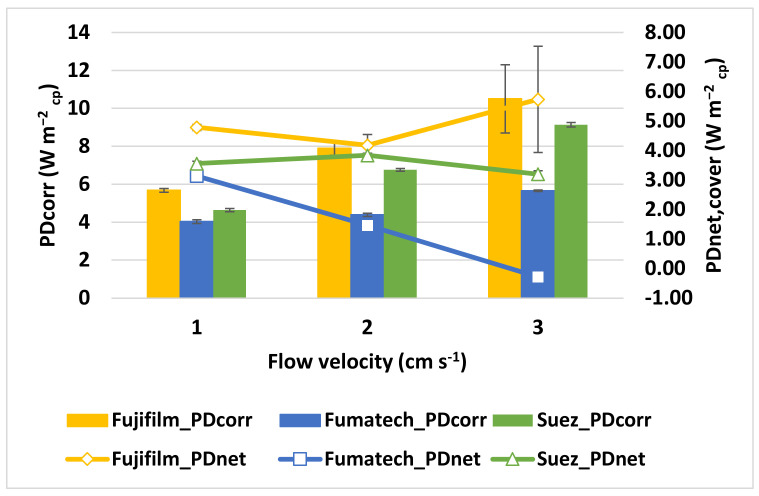
Influence of fluid velocity on power density for the different configurations under the experimental conditions of 5 mol L^−1^ *C_High_*, 0.06 mol L^−1^ *C_Low_*, and 40 °C temperature.

**Figure 11:**
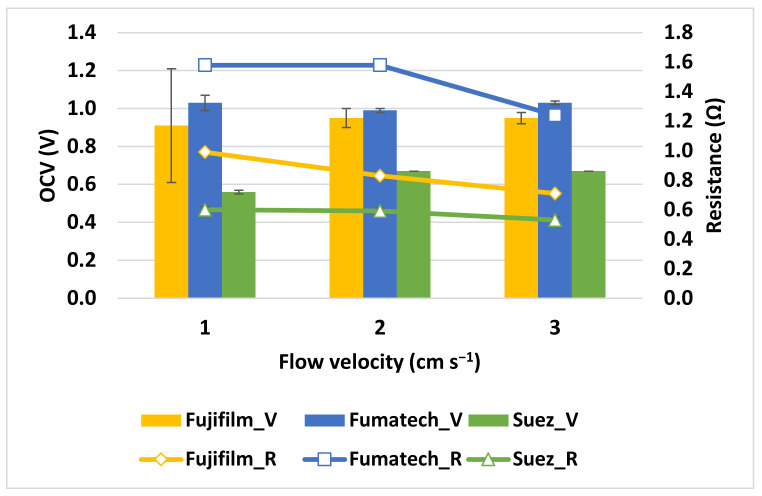
Variation of *OCV* and stack resistance with flow velocity under the experimental conditions of 5 mol L^−1^ *C_High_*, 0.06 mol L^−1^ *C_Low_*, and 40 °C temperature.

The linear flow velocity of the streams running through the RED stack is strongly linked to net power density, and therefore, a specific working range is widely suggested. In this section, the RED stack was investigated between 1 and 3 cm s^−1^. In this sense, three different IEMs were studied using a five-cell pairs stack. Figure 10 demonstrates the corrected power and net power density achieved for various flow velocities. The maximum *PD*_corr_ increases by 46% (Fujifilm stack), 29% (Fumatech stack), and 51% (Suez stack) as the velocity increases from 1 to 3 cm s^−1^, allowing it to reach almost 10.5 W m^−2^_cp_ for the Fujifilm stack. However, the increase in *PD*_corr_ from 2 to 3 cm s^−1^ is much more limited than the one obtained from 1 to 2 cm s^−1^. Even if the corresponding *OCVs* have shown modest improvement, stack internal resistance is reduced by the increased flow velocity (see Figure 11), thus improving power density. The increase of feed velocity on net power output is more evident due to the dramatic increase in hydraulic losses. In particular, the net power density becomes negative for the RED unit assembly with Fumatech IEMs, at the flow velocity of 3 cm s^−1^, whereas Fujifilm exhibits the highest net power density of 5.8 W m^−2^_cp_, and Suez showed 3.20 W m^−2^_cp._

Regardless of the type of IEMs used, identical dependencies of *OCV* and stack resistance on stream velocity were observed. *OCV*, for instance, generally increases with velocity, whereas resistance decreases with velocity (Figure 11). The *OCV* increase with velocity is due to the lower residence time in the stacks: the shortened residence duration results in a lower salinity gradient change between *C_High_* and *C_Low_* compartments and in a driving force that decreases poorly along the streamwise direction [33]. The increase in flow rate results in an increase in the salinity gradient energy entering the RED stack per unit of time. The higher *OCV*, when using higher flow velocity, may be attributed to the lower effect from concentration polarization phenomena. The output power of all the stacks assembled with different IEMs is large with a high flow rate. The excessive flow velocity will enhance the hydrodynamic loss in the stack.

Finally, the best results collected in the present work were compared with other relevant results available in the literature. As can be seen in Table 4 where all data are summarized, the power density achieved in the present work with the RED unit equipped with Fujifilm membranes (i.e., first row in the table) was the highest for feed solutions at 40 °C. Only a RED unit provided with Neosepta membranes and operated at 60 °C was able to achieve a higher power density. Of course, this temperature is difficult to obtain naturally (i.e., bittern discharged in summer); rather, a solar or waste heat recovery would be needed to achieve such power.

## 4. Conclusions

A parametric analysis on RED stacks fed with saltworks bitterns and equipped with different IEMs was performed. The influence of ion exchange membranes produced by different manufacturers, temperature, and flow velocities on the conversion of chemical energy to electrical energy was investigated.

Among the three different membranes tested in the present work, the RED unit equipped with Fujifilm Type 10 membranes has exhibited the best performance. A maximum *PD_corr_* of 5.1 W m^−2^_cp_ using 1 cm s^−1^ flow velocity at room temperature (20 °C) has been found due to the fact that this membrane combines lower resistance and high permselectivity.

It has been confirmed that temperature has a beneficial effect on power density *PD_corr_*, raising from 5.06 W m^−2^_cp_ to 6.01 W m^−2^_cp_ (Fujifilm stack) with increasing the temperature from 20 to 30 °C.

In conclusion, a maximum power density of 10.5 W m^−2^_cp_ using *C_High_* 5 mol L^−1^, *C_Low_* 0.06 mol L^−1^, and a flow velocity of 3 cm s^−1^ has been achieved in this study, when using Fujifilm type 10 IEMs.

## Figures and Tables

**Table 1:** Properties of the ion exchange membranes (IEMs) used in this work.

Membrane	Fujifilm [24]		Fumatech [25]		Suez [26]	
	AEM Type 10	CEM Type 10	AEM FAB	CEM FKB	AEM AR103U	CEM CR67U
Thickness dry (μm)	125	135	130	130	130	150
Electrical resistance (Ω cm^2^)	1.7	2.0	<8.5	<5	1.4	2.0
Permselectivity	95	99	>93	>98	90	90
IEC (m_eq_ g^−1^)	1.8	1.5	-	-	2.37	1.92
Water permeability (mL bar^−1^ m^−2^ h^−1^)	6.5	6.5	-	-	-	-

The information in Table 1 has been collected from the data sheets of membrane manufacturers, when available.

**Table 2:** Conditions selected for the experiments.

Parameter	Reference Test Value
Dilute concentration	0.01–0.1 M NaCl
Concentrate concentration	5 M NaCl
Fluid velocity	1–3 cm s^−1^
Spacer thickness (µm)	270
Temperature	20–40 °C
Effective area of membrane (m^2^)	0.01
ERS solution composition	0.1 M of FeK3(CN)6/FeK4(CN)6 and 0.6 M NaCl
Conductivity	105 mS/cm
Pumping efficiency	90%
Temperature	20–40 °C

**Table 3:** Leakage test results using deionized water in all channels.

Stack	Fujifilm	Suez		Fumatech
AEM	Type-10	AR103U		FAB-PK-130
CEM	Type-10	CR67U		FKB-PK-130
Flow velocity (cm s^−1^)	1	1		1
Internal leakage	<0.1%	HC	LC	0%
		6%	12%	

**Table 4:** Comparison of present data to those previously reported in the scientific literature.

Membrane	Experimental Conditions				Concentration (mol L^−1^)		Performance	Reference
Supplier	N° pairs	Flow velocity (cm s^−1^)	Area (cm^2^)	Temp (°C)	*C_High_*	*C_Low_*	Power density (W m^−2^)_CP_	
Fuji Type 10	5	3	10 × 10	40	5	0.06	10.5	This work
Fuji- Type II	-	8.7 mm s^−1^	-	50	3	0.6	0.26	[28]
Fumasep	50	4	10 × 10	40	5	0.5	12	[3]
Neosepta^®^	4	0.81	10 × 10	40	0.513	0.017	1.88	[33]
YDS	8	0.717	17 × 7	40	66.70 g L^−1^	0.66 g L^−1^	0.88	[34]
Fuji-Type II	10	8.55 mm s^−1^	0.118 × 0.065 m^2^	50	3	0.06	0.2	[19]
Neosepta^®^	5	25 mL min^−1^	10 × 10	60	5	0.01	13.4	[20]

## Data Availability

The data presented in this study are available on request from the corresponding author and will be uploaded online into the SEArcularMINE project Zenodo platform.

## Nomenclature and Abbreviations

**Nomenclature**	
*A*	Active membrane area (m^2^)
*I*	Electric current (I)
*N*	Number of cell pairs (-)
*OCV*	Open circuit voltage (V)
*P*	Power (W)
*PD*	Power density (W/m^2^ cell pair)
*PD_corr_*	Corrected power density (W/m^2^ cell pair)
*PD_net,cover_*	Net Covered power density (W/m^2^ cell pair)
*PD_pump_*	Pumping power density (W/m^2^)
Δ*P*	Pressure drop (Pa)
*Q*	Volumetric flow rate (mL/min)
*Q^tot^*	Total feed volumetric flow rate (m^3^/sec)
*R_blank_*	Blank resistance (Ω)
*R_cell_*	Resistance of cell pair (Ω)
*R_stack_*	Stack internal resistance (Ω)
*R_load_*	Load resistance
*R*	Real gas constant (J/mol/K)
*V_stack_*	Stack potential (V)
*T*	Temperature (K)
*V*	Fluid flow velocity (cm s^−1^)
**Abbreviations**	
AEM	Anion exchange membrane
ERS	Electrode rinse solution
IEMs	Ion exchange membrane
HCS	High compartment solution
LCS	Low compartment solution
RED	Reverse Electrodialysis
SGP	Salinity Gradient power
*z*	Valence number
